# Dysregulation of melatonin rhythm in Parkinson’s and Huntington’s disease: a systematic review and meta-analysis

**DOI:** 10.3389/fnagi.2025.1637881

**Published:** 2025-10-09

**Authors:** Reema Priyanka Suram, Rida Fatima, Rajesh Madhuvilakku, Jin Ho Jung, Sang Jin Kim, Yonggeun Hong

**Affiliations:** ^1^Department of Rehabilitation Science, Graduate School of Inje University, Gimhae, Republic of Korea; ^2^Biohealth Products Research Center (BPRC), Inje University, Gimhae, Republic of Korea; ^3^Research Center for Aged-life Redesign (RCAR), Inje University, Gimhae, Republic of Korea; ^4^Department of Physical Therapy, College of Healthcare Medical Science and Engineering, Inje University, Gimhae, Republic of Korea; ^5^Department of Neurology, Busan Paik Hospital, Inje University College of Medicine, Busan, Republic of Korea; ^6^Dementia and Neurodegenerative Disease Research Center, Inje University, Busan, Republic of Korea

**Keywords:** melatonin, Parkinson’s disease, Huntington’s disease, biomarker, diagnosis, systematic review, meta-analysis

## Abstract

**Background:**

Parkinson’s disease (PD) and Huntington’s disease (HD) are progressive neurodegenerative diseases with early non-motor symptoms, such as sleep disturbances, which often precede motor symptoms but are frequently overlooked. Although HD can be diagnosed genetically, PD lacks reliable biomarkers for its early detection. Melatonin, a circadian regulator, may be a promising early biomarker to address this issue.

**Methods:**

A database search was performed to identify relevant studies. Meta-analyses were conducted using the ratio of means (RoM) as an effect size and I^2^ as a heterogeneity test.

**Results:**

Melatonin rhythmicity was significantly disrupted in both PD and HD groups. PD patients showed reduced amplitude [RoM = 0.76, 95% CI (0.26 to 1.26); *p =* 0.00] and increased 24-h area under the curve (AUC) [RoM = 1.06, 95% CI (0.26 to 1.85); *p =* 0.01]. In manifest HD, both amplitude [RoM = 0.92, 95% CI (0.81 to 1.02); *p =* 0.00] and acrophase [RoM = 0.92, 95% CI (0.07 to 1.78); *p =* 0.03] significantly decreased. PD patients with sleep disorders had significantly higher melatonin concentrations than the non-sleep disorder group, with a significant test group difference of *p =* 0.00. HD patients showed a stage-wise decline.

**Conclusion:**

This study suggests that melatonin could serve as a biomarker for the early diagnosis of PD and to track the progression of HD, thus complementing existing diagnostic tools.

**Systematic review registration:**

CRD42024544116, https://www.crd.york.ac.uk/PROSPERO/view/CRD42024544116.

## 1 Introduction

Parkinson’s disease (PD) and Huntington’s disease (HD) are devastating neurodegenerative diseases with distinct yet overlapping characteristics. PD is the 2nd most prevalent neurodegenerative disorder (Lang et al., 2022; McGhee et al., 2013). It is characterized by the accumulation of *α*-synuclein-containing Lewy bodies (Bloem et al., 2021; Tysnes and Storstein, 2017), primarily affecting dopaminergic neurons in the substantia nigra (Kalia and Lang, 2015; Li et al., 2020). This leads to both motor and non-motor symptoms such as sleep disorders, cognitive deterioration, depression, and pain (Bloem et al., 2021). Sometimes, these non-motor symptoms can appear years before the motor symptoms (Kalia and Lang, 2015; Lee and Koh, 2015). In contrast, HD is caused by the mutant huntingtin gene (mHTT), which leads to the production of neurotoxins that damage the brain cells in the basal ganglion and cortex (Bates et al., 2015; Podvin et al., 2019; Ross et al., 2014; Ross and Tabrizi, 2011). This results in a triad of motor, cognitive, and psychiatric symptoms, with chorea often prominent in the early stages (Mörkl et al., 2016; Ross et al., 2014). Unlike.

PD and HD have clear genetic inheritance patterns that allow for their early detection (Podvin et al., 2019; Ross et al., 2014). While HD progresses through the premanifest and manifest stages, the lack of symptoms in the premanifest stage poses challenges for developing treatments (Bates et al., 2015).

While dopamine and *α*-synuclein are key protein biomarkers for PD, mHTT protein, and Cytosine, Adenine, Guanine (CAG) trinucleotide repeat expansion for HD, there is a crucial need for biomarkers that can detect these diseases before debilitating motor symptoms arise. Current biomarkers of PD primarily reflect the later stages of the disease process, by which time significant neuronal loss has already occurred (Park et al., 2019; Reed et al., 2018; Vermeiren et al., 2020). For instance, in PD, 70% of substantia nigral neurons may degenerate before motor symptoms become noticeable, often more than a year after the disease onset (Kalia and Lang, 2015; Vermeiren et al., 2020). Similarly, in HD, substantial striatal neuronal damage can occur decades before motor diagnosis (Reed et al., 2018).

Melatonin, a sleep hormone, has been associated with both PD and HD. Abnormal melatonin levels are frequently observed under both conditions (Aziz et al., 2009; Breen et al., 2014). Furthermore, correlations have been found between melatonin levels in both early and advanced stages of PD (Leston et al., 2010; Li et al., 2020). In patients with PD, some studies found an advanced nocturnal melatonin acrophase in medicated-PD (med-PD) individuals compared to healthy controls (Catala et al., 1997; Fertl et al., 1991) and *de novo* patients (Bordet et al., 2003; Fertl et al., 1993), while parameters such as mean value (mesor) and amplitude remained stable (Fertl et al., 1991). On the other hand, early-stage PD patients exhibited diminished melatonin rhythms compared to healthy controls, suggesting that melatonin disruption begins early in the disease (Breen et al., 2014; Videnovic et al., 2014b). Studies have also shown that PD patients with excessive daytime sleepiness (EDS) have lower melatonin levels, linking melatonin dysregulation with this common symptom (Videnovic et al., 2014b). Additionally, a reduced melatonin acrophase is associated with decreased rapid eye movement sleep, further emphasizing the complex interplay between melatonin and sleep architecture in PD (Breen et al., 2014). In HD, no major melatonin changes were observed in the early stages (Adamczak-Ratajczak et al., 2017; Aziz et al., 2009). However, in later stages, both acrophase and amplitude were reduced (Adamczak-Ratajczak et al., 2017; Kalliolia et al., 2014).

Given these findings, melatonin emerges as a promising candidate with diagnostic significance before the onset of motor symptoms in both PD and HD patients. There is promising but inconsistent evidence regarding melatonin’s role in PD and HD progression, emphasizing the need for further investigation (Kim et al., 2023; Li et al., 2020). Questions remain about how dopaminergic treatments impact melatonin secretion in PD (Lin et al., 2014; Videnovic et al., 2014b) and how their rhythm is different in PD patients with or without sleep disorders. Therefore, through this systematic review and meta-analysis (SRMA), we aim to explore how endogenous melatonin rhythm has been disrupted in PD and HD by examining the amplitude (half distance between the highest and lowest points of melatonin) and area under the curve (AUC), which indicates the total amount of melatonin secreted in 24 h in PD, and the amplitude and acrophase (peak level of melatonin concentration during a circadian rhythm) of melatonin rhythm in manifest HD. We also examined how melatonin levels are influenced by various factors in PD and HD, including disease stage, duration, sex, and age in PD as well as stage and CAG repeat expansion in HD. Ultimately, this research seeks to determine whether melatonin holds potential as a reliable biomarker for both diseases, with implications for diagnosis and treatment.

## 2 Methods

The SRMA was conducted in compliance with the Preferred Reporting Items for Systematic Reviews and Meta-Analyses (PRISMA) framework and upheld ethical principles for systematic review publications (Page et al., 2021; Wager and Wiffen, 2011). The study protocol was registered in the International Prospective Register of Systematic Reviews (PROSPERO) under registration number CRD42024544116 in May 2024.

### 2.1 Search strategy

In this SRMA article, reporting endogenous melatonin levels in PD or HD was included. This study incorporated an extensive literature search strategy that spanned several well-known databases, namely PubMed, Embase, ISI Web of Science, and the Cochrane Library. Various keywords, such as “Parkinson’s disease,” “Huntington’s disease,” “melatonin,” “n-acetyl-5-methoxy-tryptamine,” and “5-methoxy-n-acetyl-tryptamine,” were used as search terms. In addition, forward and backward citations of the included studies were performed to identify any similar studies. The extensive search strategy used in the above-mentioned databases is shown in Appendix 1.

### 2.2 Inclusion and exclusion criteria

Studies were required to satisfy the following PECO (Population, Exposure, Comparator, and Outcome) criteria to be included. (a) P: Individuals diagnosed with PD who satisfied the United Kingdom PD Society Brain Bank Criteria or the Chinese PD diagnostic criteria. Patients with HD are assessed using the Unified Huntington’s Disease Rating Scale (UHDRS). (b) E: Melatonin concentration obtained from the blood, urine, or saliva of PD or HD patients. (c) C: Age-matched healthy control group without mental illness or neurodegenerative disease symptoms. (d) O: Reported melatonin concentrations in both patient and control groups.

Studies that did not focus on PD or HD subjects with a corresponding healthy group, lacked melatonin level data, or presented results in formats other than the mean and standard deviation (SD), standard error of mean (SEM), or median with interquartile range (IQR) were removed from the SRMA. *In vitro* and animal studies, as well as articles that were retrieved and reviewed, were also excluded from the study.

### 2.3 Study selection

A thorough database search yielded a total of 2,196, which were imported to the reference manager EndNote 20 for duplicate removal, and later articles were imported to the Rayyan systematic review tool for further screening. A total of 631 duplicates were removed using duplicate removal. The titles and abstracts were screened manually, and 755 articles were excluded because they were conference abstracts. A further 757 studies were excluded as they were considered inappropriate for our study. And 21 studies were excluded due to irrelevant outcomes, and 12 studies were excluded due to unrelated exposure. Through a manual search of the forward and backward citations of the remaining 22 studies, we were able to find 2 more articles for this study. No restrictions were imposed on the language, region, or year of publication. Full texts of citations that were eligible for the study were obtained, and a thorough screening was performed independently by reviewer-1 (R1) and reviewer-2 (R2) before including them in the SRMA.

### 2.4 Data extraction

Following the final screening, the data were extracted from the included studies. The extracted information included details such as the author, country, publication year, type of study, sample size in both PD/HD and healthy groups, age, sex, specimen source for melatonin, the method used to measure levels, duration, and severity of PD or HD, CAG repeat expansions in HD, and outcomes. In cases where the published data were incomplete, articles were removed from the SRMA following unsuccessful efforts to contact the authors via email for additional information (Breen et al., 2016). For studies that only provided data in graphs, the GetData Graph Digitizer^1^ was used to extract the data. For the data represented as mean and SEM, SEM was converted to SD using (SD = SEM × √sample size) (Zhang et al., 2018).

### 2.5 Methodological quality assessment

In tandem with data extraction, we evaluated the quality of the included studies by applying the Joanna Briggs Institute (JBI) critical appraisal tool, which has 10 and 11 questions for case–control and cohort study designs, respectively, which helps to assess each study based on subjects, sampling, exposure, outcomes, and method of analysis (Moola et al., 2015; Munn et al., 2020). Articles were classified into risk categories based on the percentage of “yes” responses. A score greater than 70% was considered low risk, 50–69% moderate risk, and less than 50% high risk of bias (Elkamash and Abuohashish, 2021). This evaluation was conducted by R1 and R2, and any conflicts raised during this evaluation were rectified by the involvement of reviewer-3 (R3).

### 2.6 Statistical analysis

In this SRMA, we compared melatonin levels between subjects with PD or HD and the control group to detect potential differences among the groups. R1 and R2 independently extracted data from the eligible studies. To unify the different units and standardize the different methods employed, the ratio of means (RoM) was employed as a measure of effect size. We used RoM as an effect size because it provides a better interpretable relative measure (fold change) of the biomarker levels between the groups. Another advantage of this effect size is that it is scale-invariant and accommodates skewed distributions, which are common in biomarker data, and facilitates comparison across studies with different assay units compared to other effect sizes such as standardized mean difference (SMD) (Friedrich et al., 2008; Friedrich et al., 2011). RoM was determined by dividing the mean melatonin levels in the experimental groups by those in the control group and estimating the effect size. For instance, if melatonin levels are 31 pg./mL in the PD subjects and 21 pg./mL in the healthy subjects, the RoM would be 1.47 (31 ÷ 21). Each specific ratio was calculated within a single study, and a ratio above 1 indicated that the patient group had a higher concentration of protein than the control group, and vice versa (Olsson et al., 2016). Pooled standard error (SEpool) was determined using the Taylor series approach (Hemilä and Chalker, 2019). Pooled data were imported into the Stata SE 16 software.

Heterogeneity between studies was assessed by the I^2^ test using a fixed-effect model (FEM) if I^2^ was ≤ 50% (indicating low-moderate heterogeneity) and a random-effects model (REM) when I^2^ was > 50% (indicating high heterogeneity), which was performed using the restricted maximum-likelihood method (REML) (DerSimonian and Laird, 2015). FEM and REM were chosen according to variations between the included studies, such as methodological diversity. Subgroup analyses (≥2 studies) were conducted based on age, sex, disease severity, duration, medication status, time of day, sample type, and analytical method in PD, and on disease severity and CAG repeat numbers in HD. Meta-regression and leave-one-out sensitivity analysis (≥3 studies) were employed to determine the source of heterogeneity. Publication bias was examined using Egger’s test and a funnel plot (Egger et al., 1997, Sterne et al., 2001). Trim and fill methods were employed to check for missing studies; however, no missing studies were detected using this method of verifying publication bias. All the above analyses were performed using Stata SE 16 software.

## 3 Results

### 3.1 Selection of studies

**Figure 1:**
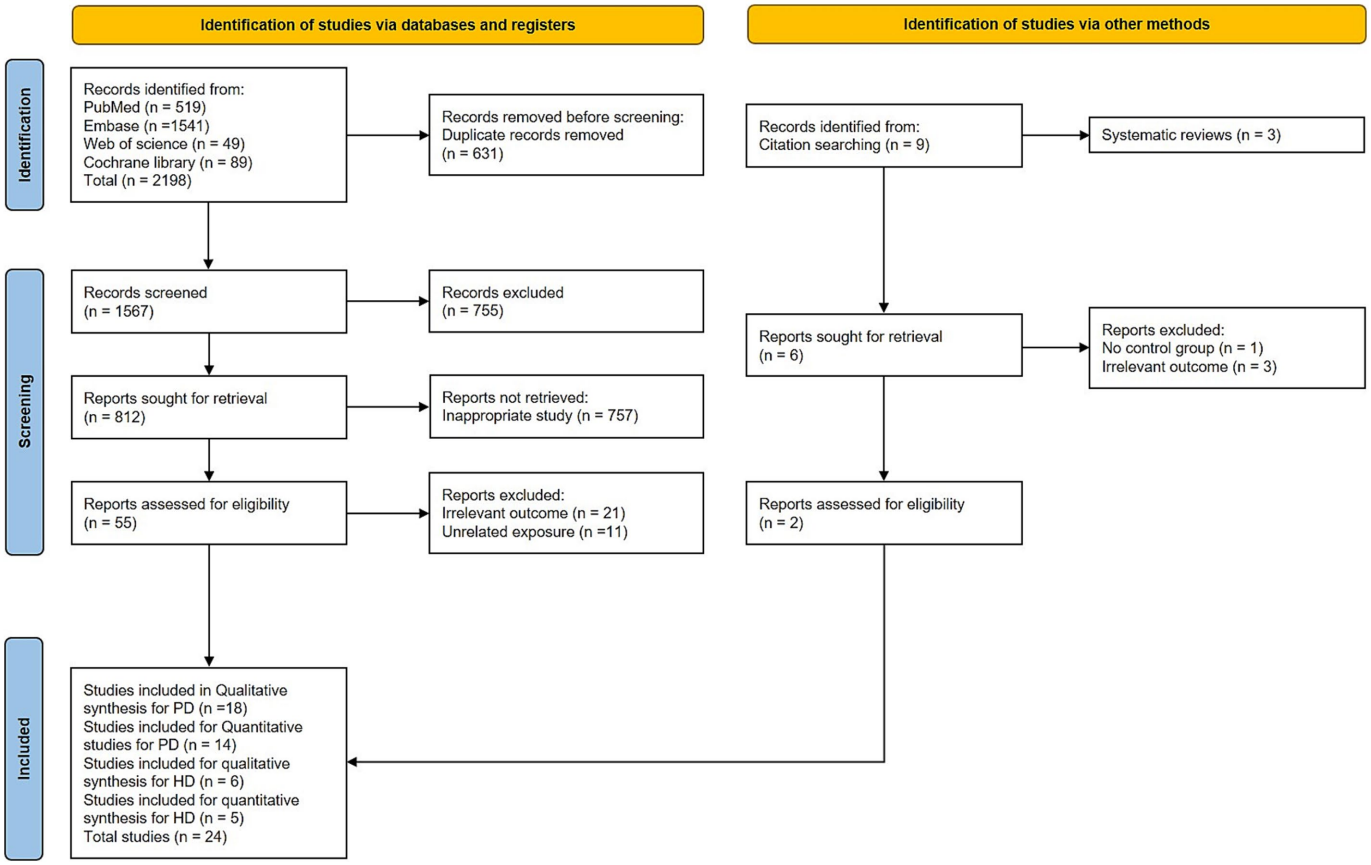
Flowchart (PRISMA 2020) for study selection.

A total of 2,198 articles were identified through a database search. After removing duplicates, 1,567 articles remained for title and abstract screening, which led to the removal of 755 articles. The remaining 812 studies were subjected to primary and secondary screenings. Of these, 757 were excluded because they are irrelevant to our study focus, 21 were excluded for irrelevant exposure, and 12 were excluded for unrelated exposure, leaving 22 studies. Additionally, two more studies were identified through manual citation searches of the included studies. Of the 24 studies, 18 were deemed eligible for the systematic review of PD, whereas 14 (Bolitho et al., 2014; Breen et al., 2014; Catala et al., 1997; Fertl et al., 1991; Fertl et al., 1993; Kataoka et al., 2020; Li et al., 2020; Li et al., 2021; Lin et al., 2014; Milanowski et al., 2023; Uysal et al., 2019; Videnovic et al., 2014b; Wei et al., 2019; Zhang et al., 2021) were included in the meta-analysis of PD. For HD, 6 studies (Adamczak-Ratajczak et al., 2017; Aziz et al., 2009; Bartlett et al., 2020; Bartlett et al., 2019; Christofides et al., 2006; Kalliolia et al., 2014) fulfilled the eligibility criteria for the systematic review, and 5 studies (Adamczak-Ratajczak et al., 2017; Aziz et al., 2009; Bartlett et al., 2019; Christofides et al., 2006; Kalliolia et al., 2014) were included in the meta-analysis. The PRISMA flowchart representing the selection of studies is shown in Figure 1.

### 3.2 Characteristics of the eligible studies

The characteristics of the eligible articles on PD and HD are detailed in Tables 1, 2, respectively. All included studies had case–control or cohort designs. Among the 18 PD studies, 16 were case–control and the remaining 2 were cohort studies, while in HD, all 6 studies were case–control studies. In the PD studies, 1,061 patients and 1,019 control subjects were analyzed. Among the PD patients, 137 (12.91%) were unmedicated, whereas 924 (87.09%) were on dopamine medication. The six HD studies included 78 healthy controls, 67 premanifest HD patients, 44 manifest HD patients, and 8 prodromal-stage HD patients. Melatonin levels from various sources have been analyzed in both PD and HD studies. Among the included studies for analysis in PD, 12 studies (66.67%) used serum, 5 studies (27.78%), and 1 study (5.56%) used saliva. And 6 studies (33.33%) analyzed 24 h, 9 studies (50%) analyzed morning, 1 analyzed habitual sleep time, 1 analyzed diurnal (11 a.m. and 12 p.m.), and 1 analyzed at 12 p.m. and 5 a.m., with 5.55% each. In HD, 2 studies used serum and plasma 40% each, and 1 study used saliva (20%), and 3 studies analyzed 24-h data (20% each). In PD, 14 studies used enzyme-linked immunosorbent assay (ELISA), and 4 studies used radioimmunoassay (RIA), and in HD, 3 studies used ELISA and 3 studies used RIA as their analytical method.

**Table 1:** Characteristics of the included studies in PD.

Study (Country)	Sample source	Method	Subjects	Design	Sample size (Case/Control)	Age (Case/Control)	Main outcome(s)
Fertl et al. (1991) (Austria)	Serum	ELISA	PD vs. Control	Case–control	9 / 14	62.1 ± 8.7 / 58.0 ± 10.4	24-h serum melatonin, AUC
Fertl et al. (1993) (Austria	Serum	ELISA	PD vs. Control	Case–control	9/14	60.0 ± 3.2 / 58.0 ± 2.8	24-h serum melatonin, AUC
Catala et al. (1997) (Spain)	Plasma	RIA	PD vs. Control	Case–control	10 / 7	—	Diurnal plasma melatonin
Bordet et al. (2003) (France)	Plasma	RIA	PD vs. Control	Case–control	26 / —	55 ± 10 / 63 ± 14	Plasma melatonin, cortisol, and temperature
Lin et al. (2014) (China)	Serum	ELISA	PD vs. Control	Case–control	56 / 22	55–90	Morning serum melatonin
Bolitho et al. (2014) (Australia)	Saliva	RIA	PD vs. Control	Case–control	28	63.6 ± 9.8 / 64.8 ± 6.0	Salivary melatonin, AUC
Videnovic et al. (2014a) (USA)	Serum	RIA	PD vs. Control	Case–control	20 / 15	64.1 ± 1.8 / 64.5 ± 1.5	Serum melatonin, AUC
Breen et al. (2014) (UK)	Serum	ELISA	PD vs. Control	Case–control	30 / 15	68 ± 9 / —	24-h serum melatonin, sleep measures
Breen et al. (2016) (UK)	Serum	ELISA	PD vs. Control	Case–control	12 / 12	66.7 ± 5.5 / 66.3 ± 5.2	24 h serum melatonin, hypothalamic volume-
Uysal et al. (2019) (Turkey)	Serum	ELISA	PD vs. Control	Case–control	40 / 40	68.3 ± 7.9 / 65.4 ± 8.3	Night serum melatonin
Wei et al. (2019) (China)	Serum	ELISA	PD vs. Control	Case–control	50/50	40–80/40–80	Serum melatonin and glutathione
Zhang et al. (2021) (China)	Serum	ELISA	PD vs. Control	Case–control	104 / 30	55.2 ± 5.1 / 55.2 ± 5.1	α-synuclein and melatonin
Kataoka et al. (2020) (Japan)	Urine	ELISA	PD vs. Control	Cohort	201 / 380	71.1 ± 7.6 / 74.3 ± 6.2	Urinary 6-sulfatoxymelatonin
Li et al. (2020) (China)	Plasma	ELISA	PD vs. Control	Case–control	61 / 58	62.4 / 64.3	Plasma melatonin
Hadoush et al. (2020) (Jordan)	Serum	ELISA	PD vs. Control	Case–control	34 / NA	NA	Serum melatonin and dopamine levels, motor, cognitive, and sleep dysfunctions
Hadoush et al. (2020	Serum	ELISA	PD vs. Control	Case–control	25 / NA	NA	Serum melatonin sleep functions, and depression levels
Li et al. (2021) (China)	Plasma	ELISA	PD vs. Control	Cohort	314 / —	66.4 ± 9.7 / 66.0 ± 9.2	Plasma melatonin, clock genes
Milanowski et al. (2023) (Poland)	Serum	ELISA	PD vs. Control	Case–control	20 / 20	68.3 ± 5.9 / 67.0 ± 2.4	Serum melatonin, adipokines

ELISA, enzyme-linked immunosorbent assay; RIA, radioimmunoassay; PD, Parkinson’s disease; NA, not available.

**Table 2:** Characteristics of the included studies in HD.

Study (Country)	Sample source	Method	Subjects	Design	Sample size (Case/Control)	Age (Case/Control)	Main outcome(s)
Christofides et al. (2006) (UK)	Serum	RIA	HD vs. Control	Case–control	11 / 15	61.6 ± 7.5 / 44.6 ± 9.0	Tryptophan metabolites, melatonin, and oxidative stress markers
Aziz et al. (2009) (Netherlands)	Plasma	RIA	HD vs. Control	Case–control	9 / 9	47.1 ± 3.4 / 48.6 ± 3.3	Diurnal melatonin levels
Kalliolia et al. (2014) (UK)	Plasma	RIA	Premanifest HD, Stage 2–3 HD	Case–control	14 / 13 / 15	45 (31–58), 55 (42–70), 52 (29–69)	Melatonin levels, acrophase, onset time
Adamczak-Ratajczak et al. (2017) (Poland)	Serum	ELISA	Early–mid HD, Late HD	Case–control	5 / 6 / 10	48.0 ± 3.0 / 45.0 ± 8.7	Serum melatonin and cortisol
Bartlett et al. (2020) (Australia)	Saliva	ELISA	Premanifest HD	Case–control	32 / 29	44.5 ± 11.4 / 44.3 ± 10.8	Salivary melatonin, cortisol, and hypothalamic volume
Bartlett et al. (2019) (Australia)	Saliva	ELISA	Premanifest and Prodromal HD	Case–control	18 / 11	50.6 ± 9.5 / NA	Salivary melatonin, cortisol, hypothalamic volume, BDNF

ELISA, enzyme-linked immunosorbent assay; RIA, radioimmunoassay; HD, Huntington’s disease; NA, not available.

### 3.3 Risk of bias and quality assessment (JBI checklist)

In Supplementary Tables 1, 2, we present the 18 studies that met the inclusion criteria for PD. All 18 studies were found to have a low risk of bias based on the JBI Checklist. Additionally, Supplementary Table 3 shows that all 6 HD studies had a low risk of bias, as evaluated using the same checklist.

### 3.4 Meta-analysis

As part of our meta-analysis of PD, we analyzed the amplitude and AUC of the 24-h melatonin rhythm.

#### 3.4.1 Alterations in endogenous melatonin rhythm in med-PD

**Figure 2:**
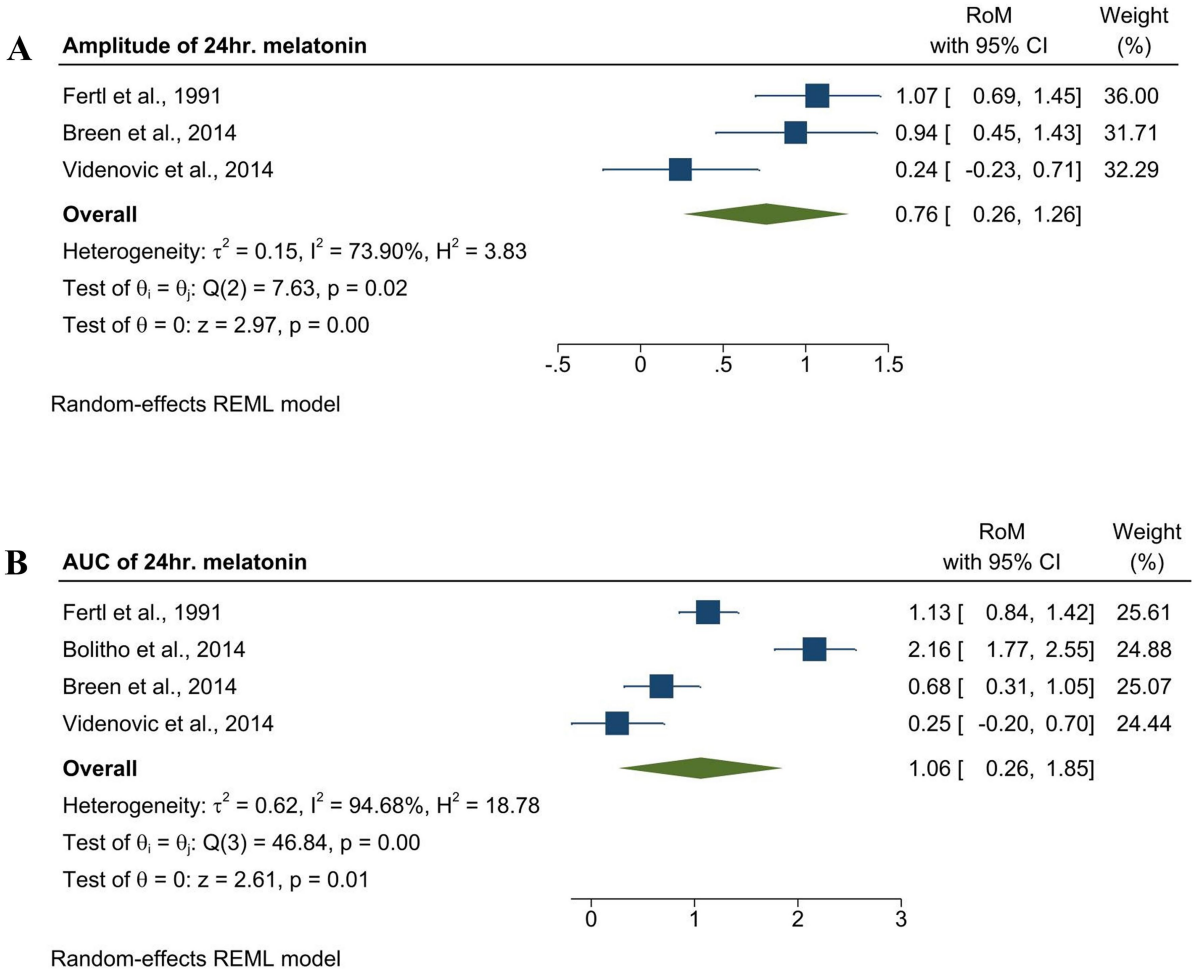
Forest plot showing the RoM of amplitude and AUC of endogenous melatonin in the med-PD and control groups. **(A)** Forest plot showing the RoM of the amplitude of melatonin in the med-PD and control groups. Individual RoMs and their corresponding 95% confidence intervals are represented by filled squares, where the size of each square denotes the weight of the study in the random-effects meta-analysis. The overall RoM estimate and its 95% confidence interval are indicated by the diamonds. **(B)** Forest plot showing the RoM of the AUC of melatonin med-PD and controls. Individual RoMs and their corresponding 95% confidence intervals are represented by filled squares, where the size of each square denotes the weight of the study in the random-effects meta-analysis. The overall RoM estimate and its 95% confidence interval are indicated by the diamonds.

To assess the changes in melatonin secretion rhythm, we analyzed the amplitude and AUC of the melatonin in the PD. Amplitude in the PD group was significantly lower compared to the control group with an effect size of [RoM = 0.76, 95% CI (0.26 to 1.26); *p =* 0.00] (Figure 2A), while AUC showed significantly elevated levels compared to the control group, as indicated by the effect size of [RoM = 1.06, 95% CI (0.26 to1.85); *p =* 0.01], as shown in Figure 2B. Both the amplitude and AUC were analyzed using the REM model.

#### 3.4.2 Comparing melatonin levels in different variables

**Figure 3:**
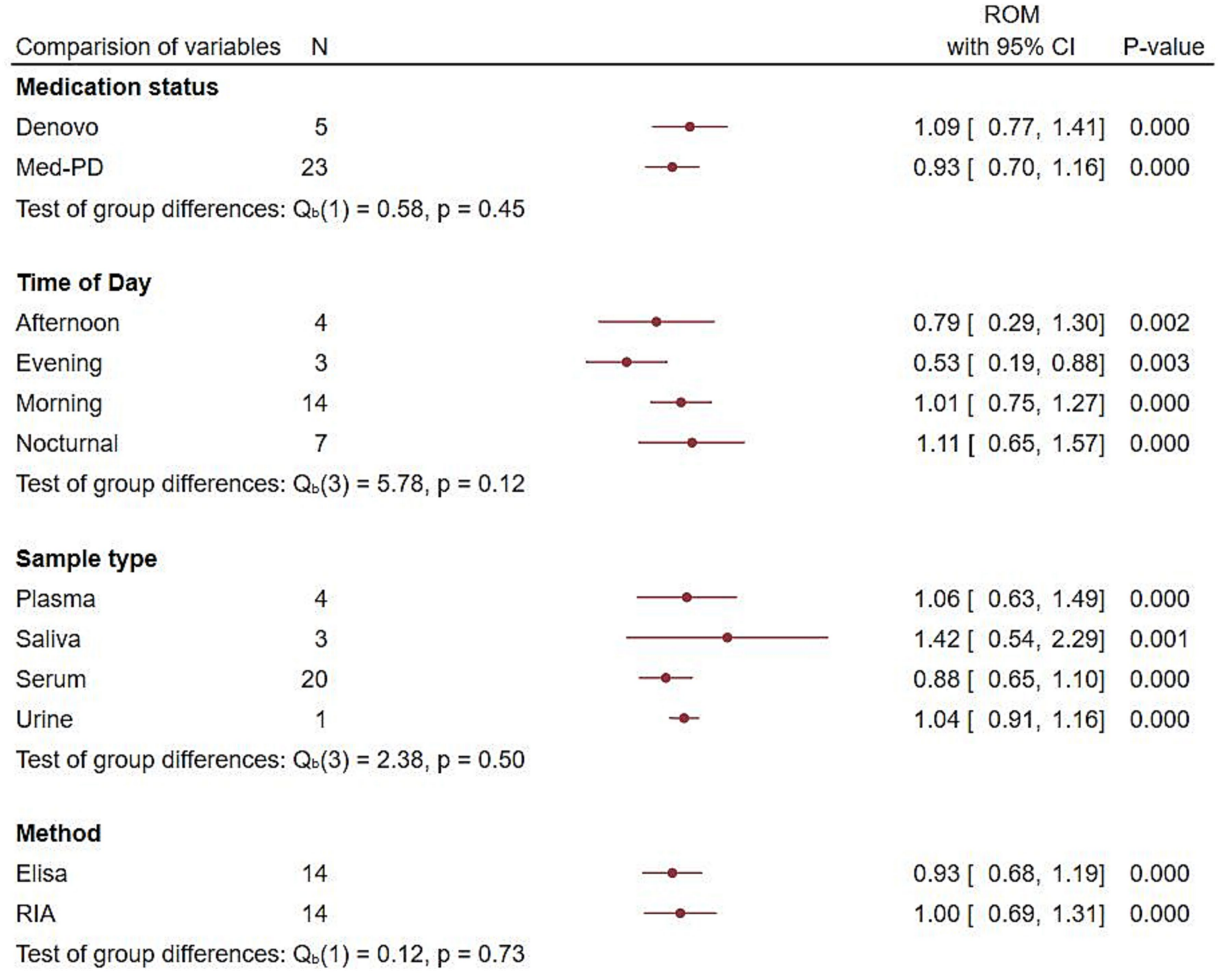
Forest plot showing endogenous melatonin alterations with different variables. Forest plot showing the RoM of alterations in endogenous melatonin based on medication status, time of day, sample type, and analytical method between the PD and control groups. RoMs and their corresponding 95% confidence intervals are represented by filled circles, with their statistical significance represented by the *p*-value.

To verify whether the variations in melatonin rhythm were consistent among the PD group compared to the control group, we performed an analysis on different variables, such as medication status, time of day, sample type, and analytical method (Figure 3). The results showed consistent variations in melatonin levels in PD patients. *De novo* patients had significantly higher melatonin levels [RoM = 1.09, 95% CI (0.77 to 1.41); *p =* 0.00], and the Med-Pd group [RoM = 0.93, 95% CI (0.70 to 2.26); *p =* 0.00] had lower melatonin levels than the controls. Morning (5 a.m.–11 a.m.) with an effect size of RoM = 1.01,95% CI (0.75 to 1.27), and nocturnal (12 a.m.–midnight) melatonin levels with RoM = 1.11, 95% CI (0.65 to 1.57) were significantly higher, whereas afternoon (12 p.m.) and evening (6 p.m.) showed considerably lower melatonin levels in the PD group. Our findings, according to sample type, showed that saliva had a stronger effect compared to other sample types, and the radioimmunoassay (RIA) method showed a stronger effect compared to the ELISA method.

#### 3.4.3 Comparing the association of melatonin levels in PD with sleeping and non-sleep disorders

**Figure 4:**
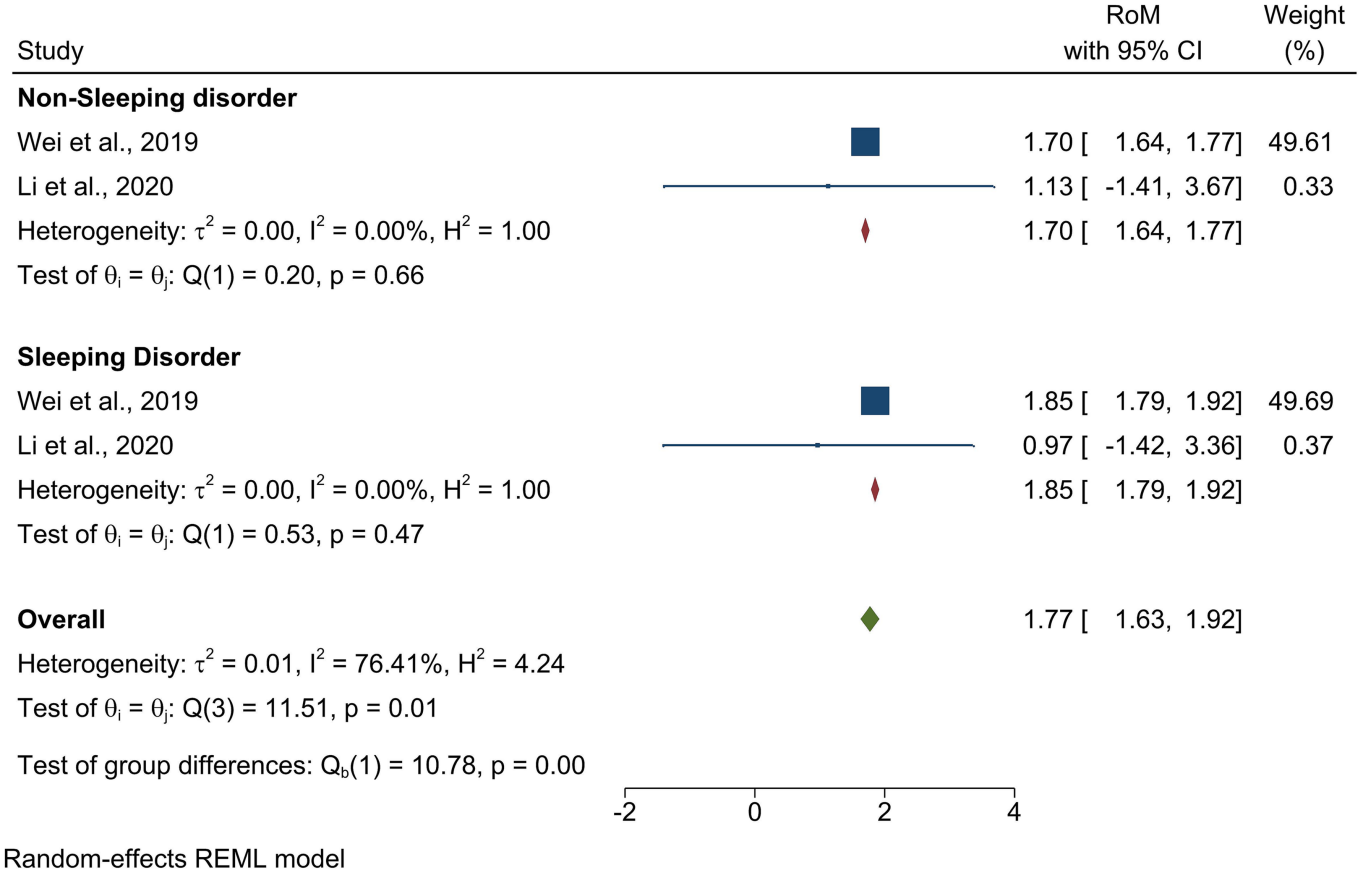
Endogenous melatonin alterations in PD patients with and without sleep disorders. The forest plot shows a comparison of the RoM of melatonin levels in PD patients with and without sleep disorders. Individual RoMs and their corresponding 95% confidence intervals are represented by filled squares, where the size of each square denotes the weight of the study in the random-effects meta-analysis. The overall RoM estimate and its 95% confidence interval are indicated by the diamonds.

Our comparative analysis of melatonin levels in PD patients with sleeping and non-sleep disorders revealed that both groups showed higher melatonin levels compared to the controls; however, the PD group with sleeping disease showed higher melatonin levels with an effect size of [ROM = 1.85, 95% CI (1.79 to 1.92)], and the test group differences showed significant differences between both the groups (*p =* 0.00) (Figure 4).

#### 3.4.4 Melatonin in HD

Analysis of HD was also included in this study, where we evaluated the melatonin rhythm based on the manifest and premanifest stages of the disease.

#### 3.4.5 Endogenous melatonin levels in the manifest stage and premanifest stage of HD

**Figure 5:**
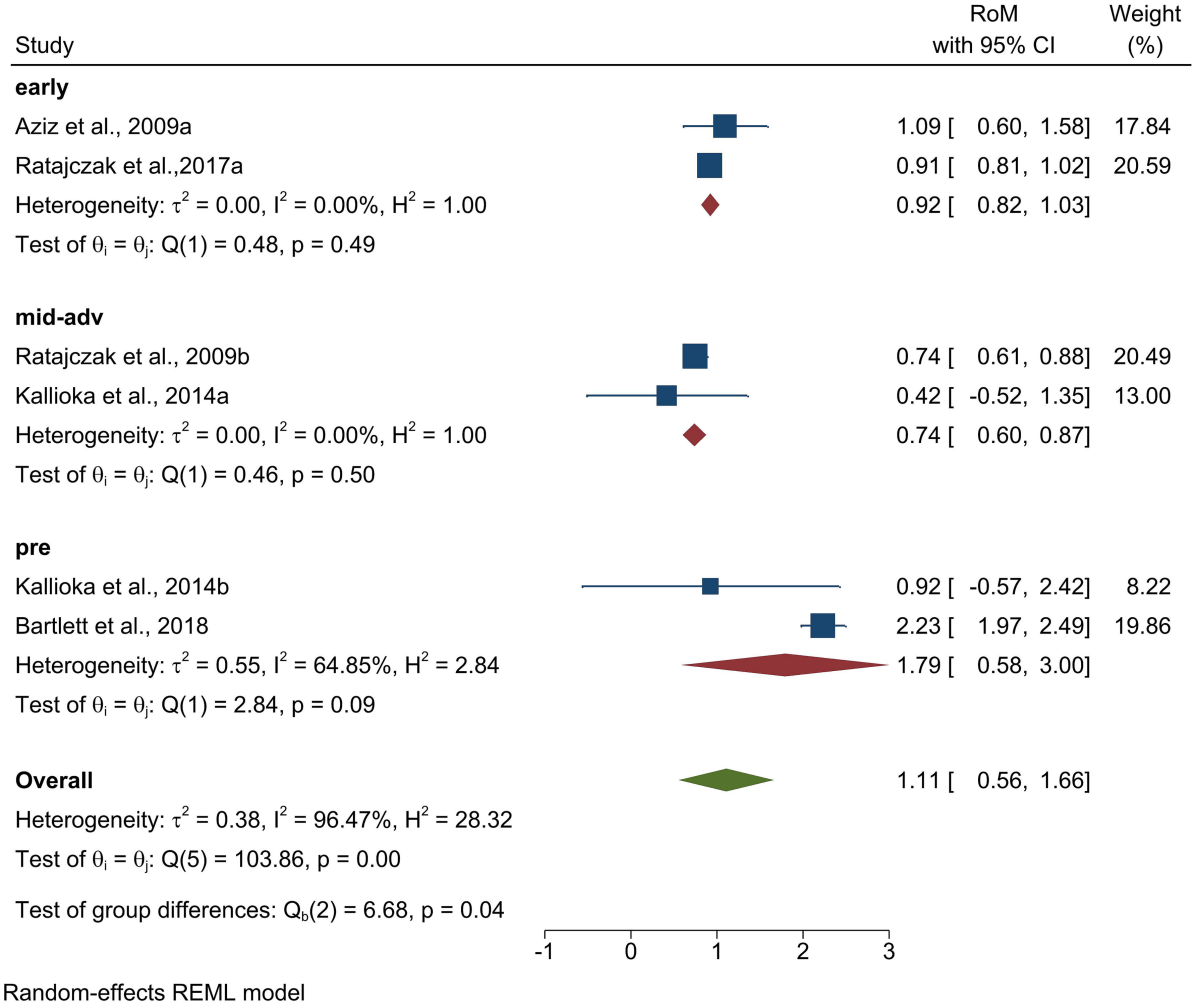
Comparing alterations of melatonin levels in Huntington’s Disease (both manifest and premanifest) patients and controls. Forest plot showing the RoM of melatonin levels between individuals with manifest HD and controls. Individual RoMs and their corresponding 95% confidence intervals are represented by filled squares, where the size of each square denotes the weight of the study in the fixed-effects meta-analysis. The overall RoM estimate and its 95% confidence interval are indicated by the diamonds.

Four studies explored the 24-h mean melatonin levels in the manifest stage of HD (Adamczak-Ratajczak et al., 2017; Aziz et al., 2009; Kalliolia et al., 2014) and two studies focused on the premanifest stage (Bartlett et al., 2019; Kalliolia et al., 2014). As shown in Figure 5, premanifest stage showed an effect size of 1.79 [RoM = 1.79, 95% CI (0.58 to 3.00)]. Manifest HD was analyzed in the early and mid-advanced stages. Both early and mid-advanced HD showed significantly lower melatonin levels compared to the controls, with effect sizes of [ROM = 0.92, 95% CI (0.82 to 1.03)] and [ROM = 0.74, 95% CI (0.60 to 0.87)], respectively. The test of group differences showed that the differences between the groups were statistically significant (*p =* 0.04).

#### 3.4.6 Alterations of melatonin rhythm in manifest HD

**Figure 6:**
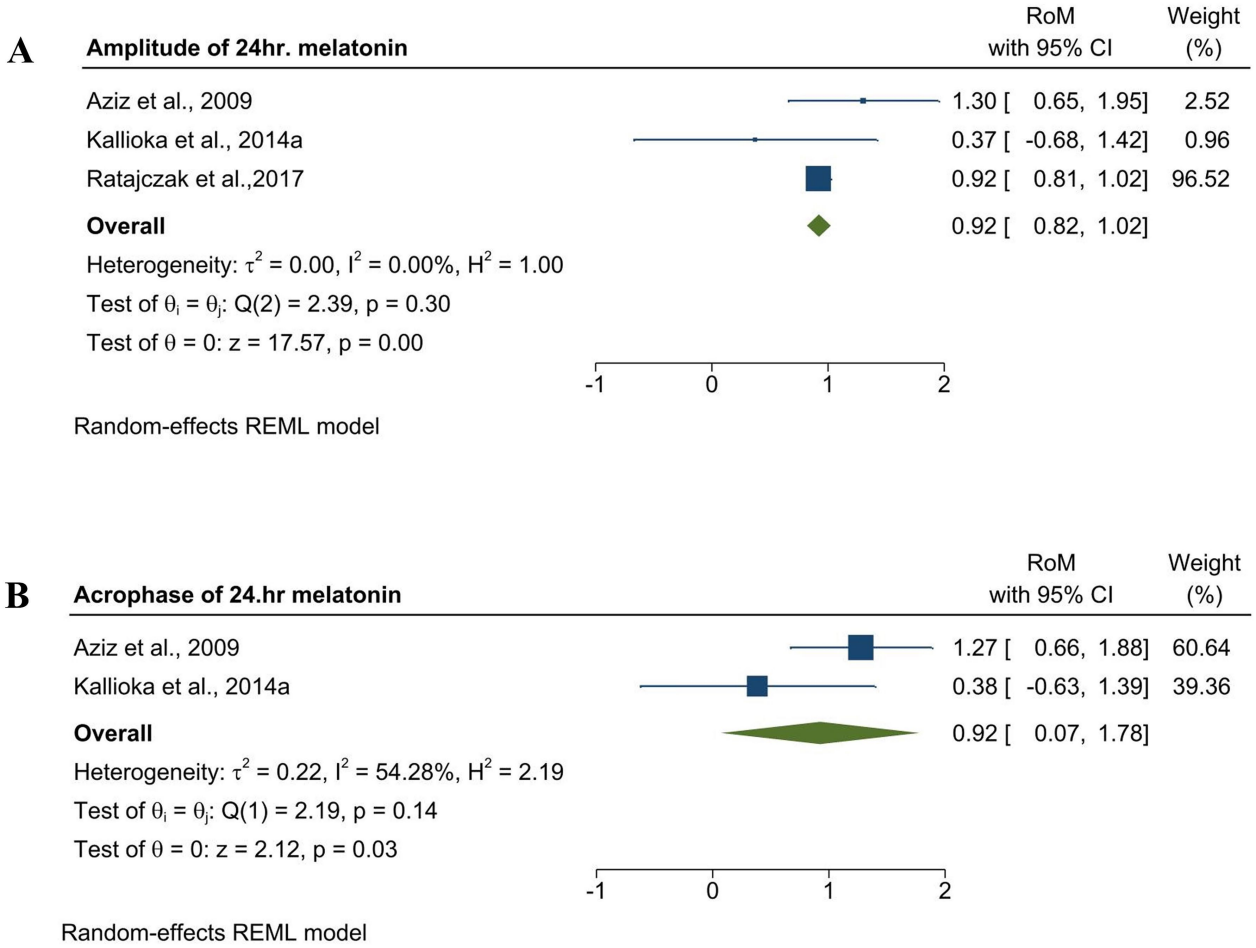
Forest plot showing the RoM of amplitude and acrophase of endogenous melatonin in med-PD and control groups. **(A)** Forest plot showing the RoM of the amplitude of melatonin in manifest HD and controls. Individual RoMs and their corresponding 95% confidence intervals are represented by filled squares, where the size of each square denotes the weight of the study in the random-effects meta-analysis. The overall RoM estimate and its 95% confidence interval are indicated by the diamonds. **(B)** Forest plot showing the RoM of acrophase of melatonin in manifest HD patients and controls. Individual RoMs and their corresponding 95% confidence intervals are represented by filled squares, where the size of each square denotes the weight of the study in the random-effects meta-analysis. The overall RoM estimate and its 95% confidence interval are indicated by the diamonds.

To verify that melatonin alterations were consistent in manifest HD, we examined changes in melatonin amplitude and acrophase in manifest HD. Both melatonin amplitude and acrophase in manifest HD were significantly lower than those in controls, with an effect size of [RoM = 0.92, 95% CI (0.82 to 1.02); *p =* 0.00] and [RoM = 0.92, 95% CI (0.07 to 1.78); *p =* 0.03], as shown in Figure 6. Both amplitude and acrophase analyses were performed using the REM model.

### 3.5 Subgroup analysis and meta-regression

#### 3.5.1 Subgroup analysis and meta-regression in PD

To evaluate the sources of heterogeneity, we performed several subgroup and meta-regression analyses. Subgroup analysis was performed based on sex and severity using the Hoehn–Yahr (H–Y) scale, Unified Parkinson’s Disease Rating Scale (UPDRS), duration of PD, and levodopa equivalent daily dosage. Gender-based subgroup analysis revealed that overall heterogeneity remained high; however, individual heterogeneity was moderate in the female group (I^2^ = 42.07%), and in men, I^2^ was zero, which indicates that gender could be a possible source of heterogeneity. The comparative results showed that women had a stronger effect than men, with significant test differences (*p =* 0.04), as indicated in Supplementary Figure 1. The other subgroup analyses also showed significant heterogeneity. Thus, we can conclude that differences in severity, duration of PD, and drug dosages could also be reasons for the high heterogeneity (Supplementary Figures 2–5). To examine the correlation between the age of PD patients and melatonin levels, we performed a meta-regression analysis, which showed a negative relationship between melatonin levels and age (*p =* 0.02) (Supplementary Figure 6). The analysis also revealed that differences in the age of the participants might be a source of heterogeneity (I^2^ = 88.84).

#### 3.5.2 Subgroup analysis in HD

Subgroup analysis based on the number of CAG repeats showed high heterogeneity, both in CAG repeats up to 42 and CAG repeats over 42, but the differences between the groups were not statistically significant (*p =* 0.30) (Supplementary Figure 7).

### 3.6 Sensitivity analysis

We conducted a sensitivity test using a leave-one-out method to examine the potential involvement of individual studies in heterogeneity and effect sizes and to check whether the overall results were affected by any individual study.

This analysis revealed that removing the Fertl et al. (1991) study from the amplitude and AUC of melatonin levels in med-PD changed the effect size to non-significant (with *p =* 0.09 and 0.07, respectively), indicating that this study had a significant effect on the overall effect size (Supplementary Tables 4, 5). Excluding Breen et al., this study found that the effect size was insignificant (*p =* 0.11). Interestingly, excluding Videnovic et al. (2014a) analysis nullified the heterogeneity and made the effect size significant (*p =* 0.00, I^2^ = 0.00%) in the amplitude of melatonin levels in PD. This result could be attributed to the use of a different sample (plasma).

Similarly, a sensitivity analysis conducted on the amplitude of endogenous melatonin levels in manifest HD showed that no single study was responsible for the overall results (Supplementary Table 6).

### 3.7 Publication bias

To evaluate the risk of publication bias, we conducted Egger’s test. Egger’s test for amplitude and AUC of melatonin levels in med-PD and amplitude of manifest HD (Appendix 2).

## 4 Discussion

This study aimed to quantitatively review the endogenous melatonin levels in PD and HD patients. We have systematically reviewed the available literature concerning melatonin levels in PD and HD, confirming altered melatonin levels that can be directly linked with non-motor symptoms of both diseases, such as sleep disorders.

Prior to our study, there were inconsistencies in the amplitude and AUC of melatonin in med-PD. Fertl et al., in their two consecutive studies, and Breen et al., in their study on early PD, concluded that there were no significant differences between the amplitude and AUC between med-PD and controls, but there was a phase advance of the melatonin peak in the med-PD group (Breen et al., 2014; Fertl et al., 1991). Another study reported that PD patients with EDS had a lower amplitude and AUC than PD patients without EDS (Videnovic et al., 2014b). However, our results showed that the overall amplitude was lower but the AUC was higher in the mid-PD group when compared to the control group. This finding aligns with a study that compared med-PD with *de novo* and control groups and concluded that the AUC in med-PD is twice that in the *de novo* and control groups (Bolitho et al., 2014). Similarly, Bordet et al., in their comparative study of med-PD and *de novo* patients, stated that there was an elevated morning AUC but a reduced nocturnal AUC in med-PD. This suggests that alterations in melatonin rhythm occur in the early stages of PD, and these alterations are worsened by sleep disorders such as EDS.

Our time point analysis presents an intriguing paradox showing higher morning melatonin levels in PD patients compared to controls, despite the expected dip in melatonin during daylight hours (Reiter et al., 2020). The results also showed markedly higher nocturnal melatonin levels in the PD group. One possible explanation for this is the influence of dopaminergic medication on pineal function. Certain dopamine-based therapies, such as levodopa and dopamine agonists, have been reported to modulate melatonin production and secretion (Santanavanich et al., 2003). However, this evidence is not entirely consistent. Some studies suggest that these treatments may contribute to elevated nocturnal melatonin levels without significantly altering daytime synthesis, potentially due to the inhibitory effects of catecholamines on daytime secretion (Bordet et al., 2003). Furthermore, *β*-adrenergic innervation of pinealocytes provides a plausible pathway for dopaminergic therapies to indirectly affect melatonin synthesis, although this response appears to occur predominantly at night (Fertl et al., 1991; Pandi-Perumal et al., 2008). This observation of elevated morning melatonin levels in med-PD raises the possibility of a connection with the worsening of PD symptoms commonly reported in the morning (Lin et al., 2014; Atkinson et al., 2005; Li et al., 2020).

The traditional biomarkers of PD largely reflect dopaminergic dysfunction and structural neurodegeneration. Cerebrospinal fluid (CSF) dopamine and its major metabolite homovanillic acid have been investigated as indices of nigrostriatal loss (Kremer et al., 2021). However, their diagnostic and prognostic value is limited owing to the variability introduced by diet and stress. More recently, *α*-synuclein has been explored as a pathophysiological marker, with reduced CSF levels reported in PD; however, its diagnostic accuracy remains moderate (AUC = 0.73) and is constrained by low specificity across other synucleinopathies (Gao et al., 2015). NfL profoundly correlates with neuroaxonal injury and aligns with disease severity and progression, including motor decline and survival, in early PD (Bäckström et al., 2020; Mollenhauer et al., 2020). In comparison with these established biomarkers, melatonin has a distinct clinically relevant domain. Rather than reflecting neurotransmitter depletion, protein depletion, or structural degeneration, melatonin secretion directly indexes circadian and sleep-wave physiology, which are highlighted to be profoundly disrupted in PD and HD and impact the patient’s quality of life. Furthermore, melatonin levels are modulated by dopaminergic therapy (Bordet et al., 2003; Videnovic et al., 2014a), suggesting that it captures both disease- and treatment-related aspects of circadian regulation.

A staggering 98% of PD patients experience at least one non-motor symptom a decade before diagnosis (Radad et al., 2023). Among the plethora of non-motor symptoms, sleep disorders such as insomnia, EDS, and rapid eye movement sleep behavior disorder (RBD) are relatively common (Claassen and Kutscher, 2011; Abbott et al., 2005). This is where melatonin, often addressed as the “sleep hormone,” takes center stage. To verify this, we performed an analysis of PD groups with and without sleep disorders, and our results showed that both groups had elevated melatonin levels, while the group with sleep disorders showed even more pronounced elevation. Thus, we can conclude that disrupted melatonin rhythm is found in PD patients with or without sleep disorders. Studies have linked melatonin dysregulation with EDS, REM sleep disorders, and overall sleep dysfunction, not only in the late stages but also in the early stages of PD (Breen et al., 2014; Hadoush et al., 2020; Videnovic et al., 2014b). Interestingly, a positive correlation has been observed between melatonin output and a reduction in hypothalamic gray matter volume, a marker of neuronal loss, in PD, which is eventually linked to disturbed sleep patterns (Breen et al., 2016). Compelling evidence linking melatonin to various symptoms, such as disturbed sleep (Zuzuárregui and Ostrem, 2020) and severity of PD (Hadoush et al., 2021; Lin et al., 2014), along with its interplay with other crucial biomarkers such as dopamine and α-synuclein, positions melatonin as a potential biomarker candidate (Hadoush et al., 2020).

While melatonin synthesis is influenced by complex molecular mechanisms, our subgroup analysis revealed that melatonin levels vary according to the sex of the patient. Prior to our study, some studies confirmed no significant differences in plasma or serum melatonin levels in relation to sex in PD patients (Li et al., 2020; Wei et al., 2019; Lin et al., 2014). Our study contradicts this issue by showing that melatonin levels are higher in females than in males in patients with PD (Supplementary Figure 1). Meta-regression analysis of PD suggests a significant negative correlation between melatonin levels and age.

A meta-analysis performed on HD showed that both early and mid-advanced stages of manifest HD showed a significant decrease, and premanifest HD showed elevated melatonin levels than the control group. This finding is consistent with a study on plasma melatonin levels in HD patients, where they noticed diminished acrophase and amplitude in the mid-advanced group of HD patients than in healthy controls (Kalliolia et al., 2014). This finding correlates with our amplitude and acrophase analysis of melatonin in manifest HD (Figure 6). In addition, our analysis of premanifest HD showed elevated melatonin levels compared with the control group. This aligns with a study showing that hypothalamic changes occur from the premanifest stages, leading to dysregulation of circadian hormones (Bartlett et al., 2019). Contradicting studies have found significant phase changes in melatonin observed only in the advanced stages of HD (Adamczak-Ratajczak et al., 2017). Although our findings provide clarity by pooling existing data, further studies should be conducted in both the manifest and premanifest stages to avoid this confusion. Furthermore, studies have found circadian disruption in the initial stages of HD and stated that melatonin levels may gradually reduce as the disease progresses, which supports our findings (Aziz et al., 2009). Corresponding to our analysis, which showed significant changes in melatonin rhythm, we suggest that it could serve as a biomarker for HD, which is also supported by a study in which they mentioned that melatonin can be a possible marker to track the course of disease progression (Kalliolia et al., 2014).

In summary, our meta-analysis provides evidence that melatonin secretion is altered in both PD and HD, reflecting circadian rhythm disruption that is closely tied to non-motor symptoms such as sleep disorders. Unlike traditional biomarkers that primarily capture dopaminergic loss, protein aggregation, and neurodegeneration, melatonin uniquely represents the circadian and sleep–wake domains, which are central to patient quality of life. Although dopaminergic medications may contribute to melatonin alterations, the precise mechanisms remain uncertain, underscoring the need for controlled longitudinal studies. Taken together, our findings support the potential use of melatonin as a complementary biomarker for tracking disease-related circadian dysfunction and its treatment effects in PD and HD. Future research should aim to clarify its mechanistic underpinnings, establish standardized sampling protocols, and evaluate its prognostic and therapeutic relevance in larger, well-characterized cohorts.

### 4.1 Limitations

Although this SRMA provides key insights into melatonin as a potential early biomarker for PD and HD, several limitations should be acknowledged. Despite an extensive database search, some eligible studies may have been missed, and a few reports presented data in formats unsuitable for inclusion in our analysis. Furthermore, most studies have assessed melatonin levels in blood samples, limiting our ability to evaluate alternative sampling methods, such as saliva or CSF. Another important limitation is the variability in assay techniques across studies, which may have introduced heterogeneity in the measured melatonin concentrations. In addition, melatonin secretion is highly sensitive to circadian phase, light exposure, and environmental conditions, which were not consistently controlled for or reported in the included studies. Most of the available literature consisted of case–control studies with relatively small sample sizes, which may increase the risk of bias and limit generalizability. Finally, the lack of longitudinal follow-up in most studies restricts insights into dynamic changes in melatonin rhythms over the course of disease progression.

## 5 Conclusion

This SRMA indicates that melatonin alterations may occur in PD and HD and could reflect underlying circadian disturbances relevant to disease pathology. Nonetheless, the current evidence remains preliminary and is shaped by heterogeneous methodologies, small cohorts, and limited follow-up data. At this stage, melatonin cannot yet be considered a reliable clinical biomarker; however, it is a promising candidate that requires further validation. To advance the field, large, longitudinal, and multicenter studies with standardized sampling and assay methods will be essential to clarify its role in disease onset, progression, and treatment response.

## Data Availability

The original contributions presented in the study are included in the article/Supplementary material, further inquiries can be directed to the corresponding author.

## Footnotes

^1^ http://getdata-graph-digitizer.com/

## Glossary

PD: Parkinson’s disease
HD: Huntington’s disease
RoM: Ratio of Mean
CSF: Cerebrospinal fluid
ELISA: Enzyme-linked immunosorbent assay
RIA: Radioimmunoassay
EDS: Excessive daytime sleepiness
RBD: Rapid eye movement sleep behavior disorders
REML: restricted maximum likelihood method
FEM: Fixed effects model
REM: Random effects model
H-Y stage: Hoehn-Yahr stage
UPDRS: unified Parkinson’s disease rating scale
UHDRS: unified Huntington’s Disease rating scale
AUC: Area under the Curve
CAG trinucleotide: Cytosine, Adenine, Guanine trinucleotide
PRISMA: Preferred reporting items for systematic reviews and meta-analyses
PROSPERO: International prospective register of systematic reviews
JBI: Joanna Briggs institute
SD: Standard deviation
SEM: Standard error of Mean
IQR: Interquartile range
med-PD: Medicated Parkinson disease
R1: Reviewer 1
R2: Reviewer 2
R3: Reviewer 3
NfL: Neurofilament light protein
