# Supplementary material for: Dysregulation of melatonin rhythm in Parkinson’s and Huntington’s disease: a systematic review and meta-analysis
Source: Front Aging Neurosci. 2025 Oct 9;17:1637881. doi: 10.3389/fnagi.2025.1637881 (PMC12546346; doi:10.3389/fnagi.2025.1637881)
Supplement: Supplementary file 1 [file Data_Sheet_1.docx]

**Supplementary Data:**

**Contents**

**1. Supplementary Tables**

**Supplementary Table 1.** Quality assessment of the included studies using the JBI critical appraisal tool for case-control studies for PD.

**Supplementary Table 2.** Quality assessment of the included studies using the JBI critical appraisal tool for cohort studies for PD.

**Supplementary Table 3.** Quality assessment of the included studies using the JBI critical appraisal tool for case-control studies for HD

**Supplementary Table 4.** Leave-one-out sensitivity analyses on amplitude of endogenous melatonin levels in medicated PD to investigate the source of heterogeneity.

**Supplementary Table 5.** Leave-one-out sensitivity analyses on area under the curve of endogenous melatonin levels in medicated PD to investigate the source of heterogeneity

**Supplementary Table 6.** Leave-one-out sensitivity analyses on amplitude of endogenous melatonin levels in manifest HD to investigate the source of heterogeneity

**2. Supplementary Figures**

**Supplementary Figure. 1** Forest Plot showing the Ratio of Mean (RoM) of endogenous melatonin levels based on gender of patients with Parkinson’s disease and healthy controls.

**Supplementary Figure. 2.** Forest Plot showing the Ratio of Mean (RoM) of endogenous melatonin levels based on severity of PD (Hoehn-Yahr stage) with Parkinson’s disease and healthy controls.

**Supplementary Figure. 3.** Forest Plot showing the Ratio of Mean (RoM) of endogenous melatonin levels based on severity of PD (Unified PD rating score- UPDRS) with Parkinson’s disease and healthy controls.

**Supplementary Figure. 4.** Forest Plot showing the Ratio of Mean (RoM) of endogenous melatonin levels based on duration of PD with Parkinson’s disease and healthy controls.

**Supplementary Figure. 5.** Forest Plot showing the Ratio of Mean (RoM) of endogenous melatonin levels based on Levodopa equivalent daily dosage (LEDD) with Parkinson’s disease and healthy controls.

**Supplementary Figure. 6.** Meta-regression correlating age of disease with melatonin levels in PD Patients.

**Supplementary Figure. 7.** Forest plot showing the RoM of melatonin levels in HD patients based on the number of CAG repeats.

**3. Appendix**

**Appendix-1.** Publication bias using Egger’s test

**Appendix-2.** Search strategy (PubMed, Embase, ISI Web of Science, Cochrane library)

**Supplementary Table 1.** Quality assessment of the included studies for PD using the JBI critical appraisal tool for case-control studies.

| **Quality scores (from 1-10)** | | | | | | | | | | | | | |
| --- | --- | --- | --- | --- | --- | --- | --- | --- | --- | --- | --- | --- | --- |
| **Items** | | | | | | | | | | | | | **Quality scores (from 1-10; higher scores indicate less risk of bias)** |
| **Study** | **Study Design** | **1** | **2** | **3** | **4** | **5** | **6** | **7** | **8** | **9** | **10** | **Overall appraisal** | **Total** |
| Fertl et al., 1991 | Case-control | Y | Y | Y | Y | Y | N | N | Y | Y | Y | I | 8 |
| Fertl et al., 1993 | Case-control | Y | Y | Y | Y | Y | N | N | Y | Y | Y | I | 8 |
| Catala et al., 1997 | Case-control | Y | Y | U | Y | Y | N | N | Y | Y | Y | I | 7 |
| Lin et al., 2013 | Case-control | Y | Y | Y | Y | Y | N | N | Y | Y | Y | I | 8 |
| Bolitho et al., 2014a | Case-control | Y | Y | Y | Y | Y | N | N | Y | Y | Y | I | 8 |
| Bolitho et al., 2014b | Case-control | Y | Y | Y | Y | Y | N | N | Y | Y | Y | I | 8 |
| Videnovic et al., 2014 | Case-control | Y | Y | Y | Y | Y | N | N | Y | Y | Y | I | 8 |
| Breen et al., 2014 | Case-control | Y | Y | Y | Y | Y | N | N | Y | Y | Y | I | 8 |
| Breen et al., 2016 | Case-control | Y | Y | Y | Y | Y | N | N | Y | Y | Y | I | 8 |
| Uysal et al., 2018 | Case-control | Y | Y | Y | Y | Y | N | N | Y | Y | Y | I | 8 |
| Li et al., 2020 | Case-control | Y | Y | Y | Y | Y | N | N | Y | Y | Y | I | 8 |
| Milanowski et al., 2023 | Case-control | Y | Y | Y | Y | Y | N | N | Y | Y | Y | I | 8 |
| Wei et al., 2019 | Case-control | Y | Y | Y | Y | Y | N | N | Y | Y | Y | I | 8 |
| Zhang et al., 2020 | Case-control | Y | Y | Y | Y | Y | N | N | Y | Y | Y | I | 8 |
| Bordet et al., 2003 | Case-control | Y | Y | Y | Y | Y | Y | Y | Y | Y | Y | I | 10 |
| Hadoush et al.,2020 | Case-control | Y | Y | Y | Y | Y | N | N | Y | Y | Y | I | 8 |
| Hadoush et al., 2020 | Case-control | Y | Y | Y | Y | Y | N | N | Y | Y | Y | I | 8 |

**Legends:** Item.1- Were the groups comparable other than the presence of disease in cases or the absence of disease in controls? Item. 2 - Were cases and controls matched appropriately? Item.3 - Were the same criteria used for the identification of cases and controls? Item.4 - Was exposure measured in a standard, valid and reliable way? Item.5 - Was exposure measured in the same way for cases and controls? Item.6 - Were confounding factors identified? Item.7 - Were strategies to deal with confounding factors stated? Item. 8- Were outcomes assessed in a standard, valid and reliable way for cases and controls? Item.9 - Was the exposure period of interest long enough to be meaningful? Item.10 - Was appropriate statistical analysis used?

**Abbreviations:** Y, Yes; N, No; U, Unclear; N/A, Not applicable; I, Included

**Supplementary Table 2.** Quality assessment of the included studies for PD using the JBI critical appraisal tool for cohort studies.

**Legends:** Item.1- Were the two groups similar and recruited from the same population? Item. 2 - Were the exposures measured similarly to assign people to both exposed and unexposed groups? Item.3 - Was the exposure measured validly and reliably? Item.4 - Were confounding factors identified? Item.5 - Were strategies to deal with confounding factors stated? Item.6 - Were the groups/participants free of the outcome at the start of the study (or at the moment of exposure)? Item.7 - Were the outcomes measured validly and reliably? Item. 8- Was the follow-up time reported and sufficient to be long enough for outcomes to occur? Item.9 Was follow-up complete, and if not, were the loss reasons to follow up described and explored? Item.10 - Were strategies to address incomplete follow-up utilized? Item.11 - Was appropriate statistical analysis used?

**Abbreviations:** Y, Yes; N, No; U, Unclear; N/A, Not applicable; I, Included

| **Quality scores (from 1-10)** | | | | | | | | | | | | | |  |
| --- | --- | --- | --- | --- | --- | --- | --- | --- | --- | --- | --- | --- | --- | --- |
| **Items** | | | | | | | | | | | | | | **Quality scores (from 1-11; higher scores indicate less risk of bias)** |
| **Study** | **Study Design** | **1** | **2** | **3** | **4** | **5** | **6** | **7** | **8** | **9** | **10** | **11** | **Overall Appraisal** | **Total** |
| Kataoka et al., 2020 | Cohort | Y | Y | Y | N | N | Y | Y | Y | Y | N/A | Y | I | 8 |
| Li et al., 2021 | Cohort | Y | Y | Y | N | N | Y | Y | Y | Y | N/A | Y | I | 8 |

**Supplementary Table 3.** Quality assessment of the included studies of HD using the JBI critical appraisal tool for case-control studies for HD.

| **Quality scores (from 1-10)** | | | | | | | | | | | | | |
| --- | --- | --- | --- | --- | --- | --- | --- | --- | --- | --- | --- | --- | --- |
| **Items** | | | | | | | | | | | | | **Quality scores (from 1-10; higher scores indicate less risk of bias)** |
| **Study** | **Study Design** | **1** | **2** | **3** | **4** | **5** | **6** | **7** | **8** | **9** | **10** | **Overall appraisal** | **Total** |
| Christofides et al., 2006 | Case-control | Y | Y | Y | Y | Y | N | N | Y | Y | Y | I | 8 |
| Aziz et al., 2009 | Case-control | Y | Y | Y | Y | Y | N | N | Y | Y | Y | I | 8 |
| Kalliolia et al., 2014a | Case-control | Y | Y | Y | Y | Y | N | N | Y | Y | Y | I | 8 |
| Kalliolia et al., 2014b | Case-control | Y | Y | Y | Y | Y | N | N | Y | Y | Y | I | 8 |
| Ratajczak et al., 2017 | Case-control | Y | Y | Y | Y | Y | N | N | Y | Y | Y | I | 8 |
| Bartlett et al., 2018 | Case-control | Y | Y | Y | Y | Y | N | N | Y | Y | Y | I | 8 |
| Bartlett et al., 2019 | Case-control | Y | Y | Y | Y | Y | N | N | Y | Y | Y | I | 8 |

**Legends:** Item.1- Were the groups comparable other than the presence of disease in cases or the absence of disease in controls? Item. 2 - Were cases and controls matched appropriately? Item.3 - Were the same criteria used for the identification of cases and controls? Item.4 - Was exposure measured in a standard, valid and reliable way? Item.5 - Was exposure measured in the same way for cases and controls? Item.6 - Were confounding factors identified? Item.7 - Were strategies to deal with confounding factors stated? Item. 8- Were outcomes assessed in a standard, valid and reliable way for cases and controls? Item.9 - Was the exposure period of interest long enough to be meaningful? Item.10 - Was appropriate statistical analysis used?

**Abbreviations:** Y, Yes; N, No; U, Unclear; N/A, Not applicable; I, Included

**Supplementary Table 4.** Leave-one-out sensitivity analyses on amplitude of endogenous melatonin levels in medicated PD to investigate the source of heterogeneity.

|  | **The ratio of Mean [95%CI]** | **Between-study heterogeneity** |
| --- | --- | --- |
| **Overall** | 0.76(0.26 to 1.26) | *p* = 0.00; I^2^ = 73.90% |
| **Omitted study** | | |
| Fertl et al., 1991 | 0.59(-0.10 to 1.27) | *p* =0.09; I^2^ = 75.36% |
| Breen et al., 2014 | 0.67(-0.14 to 1.48) | *p* = 0.11; I^2^ = 86.10% |
| Videnovic et al., 2014 | 1.02(0.72 to 1.32) | *p* = 0.00; I^2^ = 0.00% |

**Supplementary Table 5.** Leave-one-out sensitivity analyses on Area under the Curve of endogenous melatonin levels in medicated PD to investigate the source of heterogeneity.

|  | **The ratio of Mean [95%CI]** | **Between-study heterogeneity** |
| --- | --- | --- |
| **Overall** | 1.06(0.26 to 1.85) | *p* = 0.01; I^2^ = 94.68% |
| **Omitted study** | | |
| Fertl et al., 1991 | 1.03(-0.13 to 1.85) | *p* =0.07; I^2^ = 95.81% |
| Bolitho et al., 2014 | 0.71(0.21 to 1.21) | *p=*0.01; I^2^ = 82.21% |
| Breen et al., 2014 | 1.81(0.11 to 2.26) | *p* = 0.03; I^2^ = 96.00% |
| Videnovic et al., 2014 | 1.32(0.47 to 2.17) | *p* = 0.00; I^2^ = 94.45% |

**Supplementary Table 6.** Leave-one-out sensitivity analyses amplitude of endogenous melatonin levels in manifest HD.

|  | **The ratio of Mean [95%CI]** | **Between-study heterogeneity** |
| --- | --- | --- |
| **Overall** | 0.92(0.82 to 1.02) | *p* = 0.00; I^2^ = 16.23% |
| **Omitted study** | | |
| Aziz et al., 2007 | 0.92(0.73 to 1.07) | *p* =0.00; I^2^ = 3.19% |
| Ratajczak et al., 2017 | 0.97(0.71 to 1.23) | *p* = 0.00; I^2^ = 24.07% |
| Kalliokia et al., 2014 | 0.93(0.04 to 1.82) | *p* = 0.00; I^2^ = 54.37% |

**1. Supplementary Figures**

**Supplementary Figure 1.** Forest Plot showing the Ratio of Mean (RoM) of endogenous melatonin levels based on gender in Parkinson’s disease and healthy controls. Individual RoM and their corresponding 95% CI are indicated by filled squares. The size of the square indicates the weight of the study in the random-effect meta-analysis. The summary estimate of RoM and its 95% CI are indicated by a diamond.


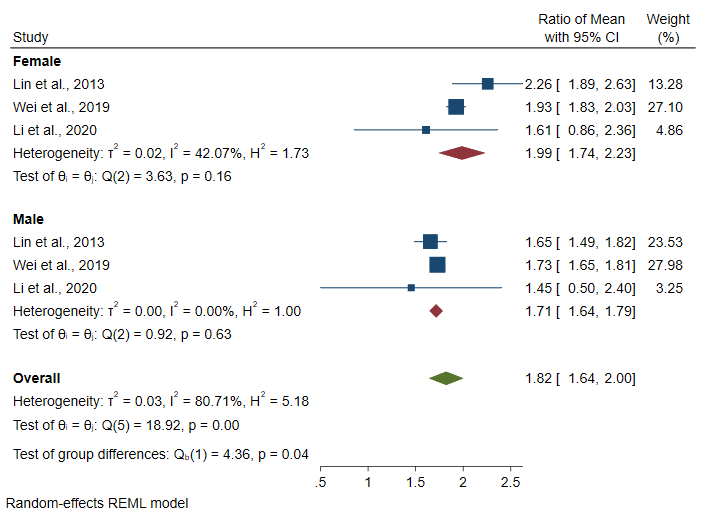


**Supplementary Figure 2.** Forest Plot showing the Ratio of Mean (RoM) of endogenous melatonin levels based on the Stage of Parkinson’s disease (Hoehn-Yahr stage). Individual RoM and their corresponding 95% CI are indicated by filled squares. The size of the square indicates the weight of the study in the random-effect meta-analysis. The summary estimate of RoM and its 95% CI are indicated by a diamond.

**
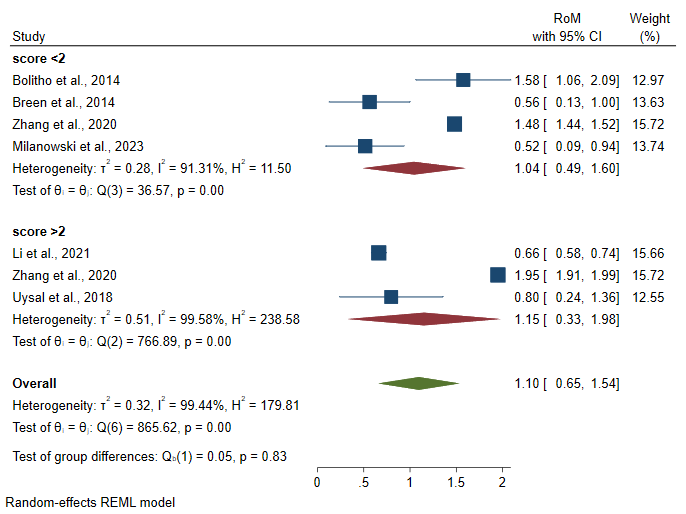
**

**Supplementary Figure 3.** Forest Plot showing the Ratio of Mean (RoM) of endogenous melatonin levels based on the severity of Parkinson’s disease (Unified PD rating scale). Individual RoM and their corresponding 95% CI are indicated by filled squares. The size of the square indicates the weight of the study in the random-effect meta-analysis. The summary estimate of RoM and its 95% CI are indicated by a diamond.


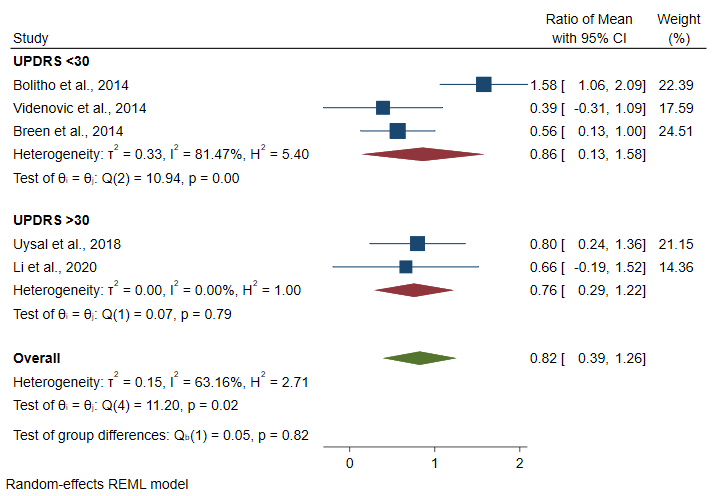


**Supplementary Figure 4.** Forest Plot showing the Ratio of Mean (RoM) of endogenous melatonin levels based on the duration of Parkinson’s disease. Individual RoM and their corresponding 95% CI are indicated by filled squares. The size of the square indicates the weight of the study in the random-effect meta-analysis. The summary estimate of RoM and its 95% CI are indicated by a diamond.


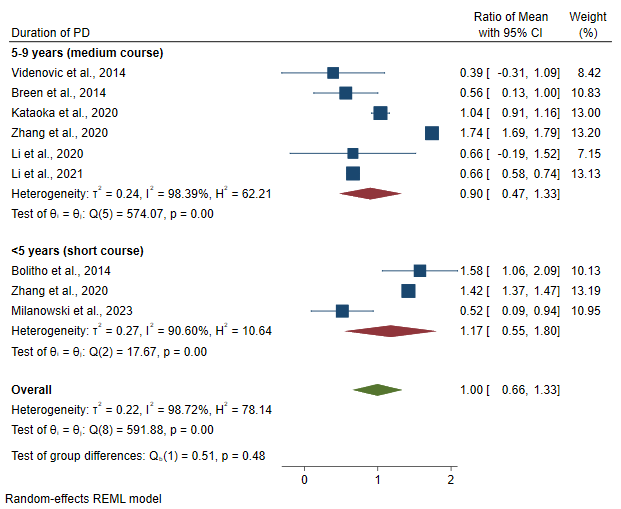


**Supplementary Figure 5.** Forest Plot showing the Ratio of Mean (RoM) of endogenous melatonin levels based on the Levodopa equivalent daily dosage(LEDD) of Parkinson’s disease. Individual RoM and their corresponding 95% CI are indicated by filled squares. The size of the square indicates the weight of the study in the random-effect meta-analysis. The summary estimate of RoM and its 95% CI are indicated by a diamond.


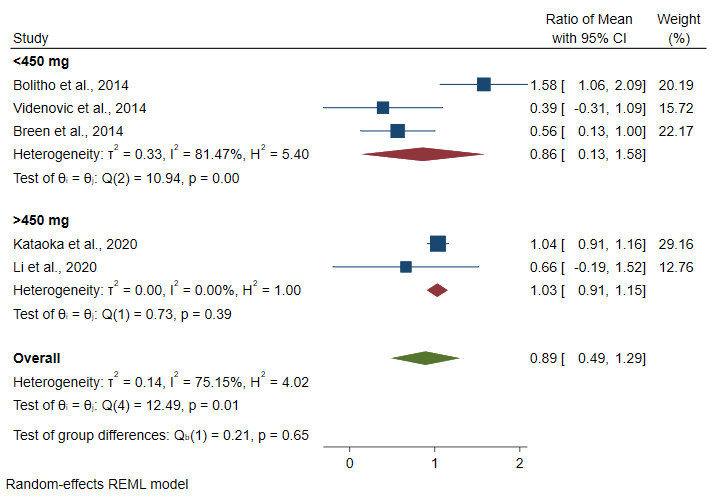


**Supplementary Figure 6.** Bubble Plot showing the RoM of endogenous melatonin levels for the age of patients with PD. The size of each circle indicates the precision of the estimate, calculated as the inverse of its within-study variance.


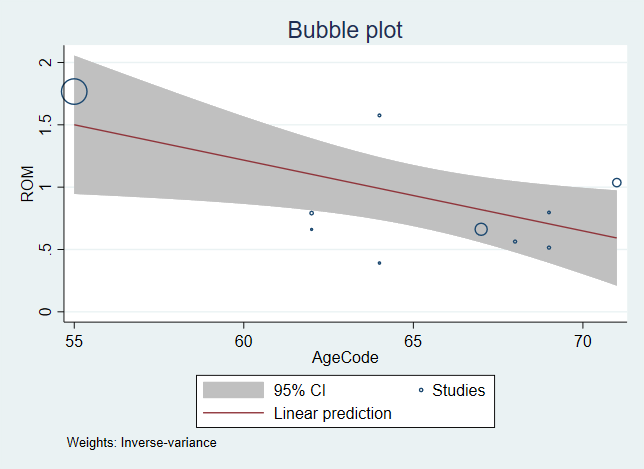


Random-effects meta-regression Number of obs = 10

Method: REML Residual heterogeneity:

tau2 = .1124

I2 (%) = 88.84

H2 = 8.96

R-squared (%) = 40.70

Wald chi2(1) = 5.06

Prob > chi2 = 0.0244

_meta_es Coef. Std. Err. z P>z [ 95% Conf. Interval]

AgeCode -.0567652 .025225 -2.25 0.024 -.1062053 -.0073251

_cons 4.6226 1.646176 2.81 0.005 1.396155 7.849046

Test of residual homogeneity: Q_res =chi2(8)=96.24 Prob > Q_res = 0.0000

**Supplementary Figure 7.** Forest plot showing the RoM of melatonin levels in HD patients based on the number of CAG repeats. The RoMs of each study, along with their 95% confidence intervals, were denoted by filled squares whose sizes correspond to their weight in the random-effects meta-analysis. The pooled estimate and its 95% confidence interval were represented by a diamond.

**
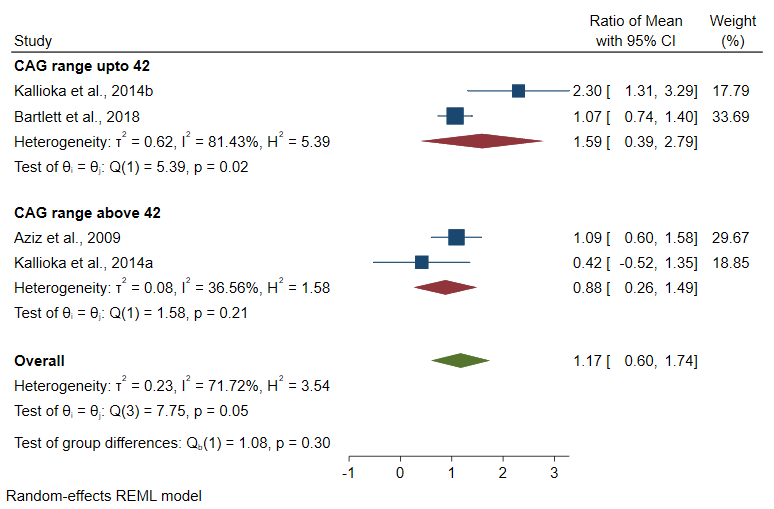
**

**3. Appendix**

**Appendix-1.** Search strategy

**SEARCH STRATEGY**

**Supplementary Table: Embase search strategy (02-05-2024)**

| **SEARCH** | **QUERY** | **ITEMS FOUND** |
| --- | --- | --- |
| **#1** | melatonin OR 'melatonin'/exp OR melatonin:ti,ab,kw OR 'n acetyl 5 methoxytryptamine'/exp OR 'n acetyl 5 methoxytryptamine':ti,ab,kw OR '5 methoxy n acetyltryptamine'/exp OR '5 methoxy n acetyltryptamine':ti,ab,kw | **50439** |
| **#2** | 'parkinson disease' OR 'parkinson disease'/exp OR 'parkinson disease':ti,ab,kw | **209735** |
| **#3** | 'huntington chorea'/exp OR 'huntington chorea' OR 'huntington disease'/exp OR 'huntington disease':ti,ab,kw | **34799** |
| **#4** | **#1 AND #2** | **1284** |
| **#5** | **#1 AND #3** | **257** |

**Supplementary Table: Cochrane Library search :02/05/2024**

|  | **QUERY** | **ITEMS FOUND** |
| --- | --- | --- |
| **#1** | (melatonin):ti,ab,kw OR (melatonin) OR (n acetyl 5 methoxytryptamine):ti,ab,kw OR (n acetyl 5 methoxytryptamine) | **4090** |
| **#2** | ("Parkinson disease"):ti,ab,kw OR ("Parkinson disease") | **13073** |
| **#3** | ("Huntington disease"):ti,ab,kw OR ("Huntington disease") | **839** |
| **#4** | **#1 AND #2** | **82** |
| **#5** | **#1 AND #3** | **7** |

**Supplementary Table: IS WEB OF SCIENCE SEARCH: 02/05/2024**

|  | **QUERY** | **ITEMS FOUND** |
| --- | --- | --- |
| **#1** | (ALL=(melatonin)) AND ALL= (parkinson disease) | **44** |
| **#2** | (ALL=(melatonin)) AND ALL= (huntington disease) | **5** |

**Supplementary Table: PUBMED search strategy (02-05-2024)**

| **SEARCH** | **QUERY** | **ITEMS FOUND** |
| --- | --- | --- |
| **#1** | **(((((((("melatonin"[MeSH Terms]) OR ("melatonin"[Text Word])) OR ("melatonin"[Title/Abstract])) OR (****5 methoxy n acetyltryptamine[MeSH Terms])) OR ("5 methoxy n acetyltryptamine"[Text Word])) OR ("5 methoxy n acetyltryptamine"[Title/Abstract])) OR (n acetyl 5 methoxytryptamine[MeSH Terms])) OR ("n acetyl 5 methoxytryptamine"[Text Word])) OR ("n acetyl 5 methoxytryptamine"[Title/Abstract])** | **33027** |
| **#2** | **((("parkinson disease"[MeSH Terms]) OR ("parkinson"[Text Word])) OR ("parkinson s disease"[Text Word])) OR ("parkinson"[Title/Abstract])** | **153886** |
| **#3** | **(("huntington disease"[MeSH Terms]) OR ("huntington disease"[Text Word])) OR ("huntington disease"[Title/Abstract])** | **15226** |
| **#4** | **#1 AND #2** | **485** |
| **#5** | **#1 AND #3** | **34** |

**Appendix-2. Publication bias using Egger’s test**

**Regression-based Egger test for small-study effects for Amplitude of melatonin in med-PD**

Random-effects model

Method: REML

H0: beta1 = 0; no small-study effects

beta1 = -7.77

SE of beta1 = 12.260

z = -0.63

Prob > |z| = 0.5263

**Regression-based Egger test for small-study effects for Area under the curve of melatonin in med-PD**

Random-effects model

Method: REML

H0: beta1 = 0; no small-study effects

beta1 = -6.09

SE of beta1 = 16.244

z = -0.37

Prob > |z| = 0.7078

**Regression-based Egger test for small-study effects for Amplitude of melatonin in manifest HD**

Random-effects model

Method: REML

Moderators: RoM SEpool

H0: beta1 = 0; no small-study effects

beta1 = -0.56

SE of beta1 = 1.595

z = -0.35

Prob > |z| = 0.7241
